# Bending Performance of Diamond Lattice Cylindrical Shells

**DOI:** 10.3390/ma18020272

**Published:** 2025-01-09

**Authors:** Sheng Li, Laiyu Liang, Ping Yang, Shaoan Li, Yaozhong Wu

**Affiliations:** 1 Wuhan Second Ship Design and Research Institute, Wuhan 430205, China; 2 Beijing Internet Based Engineering Co., Ltd., Beijing 100192, China; 3 MOTUSTECHS (WuHan) Co., Ltd., Wuhan 430073, China; 4 School of Automobile and Traffic Engineering, Wuhan University of Science and Technology, Wuhan 430081, China

**Keywords:** triply periodic minimal surface, lattice cylindrical shell structure, three-point bending, finite element analysis

## Abstract

The Diamond lattice cylindrical shell (Diamond LCS) was proposed by a mapping approach based on the triply periodic minimal surfaces (TPMS). The finite element models were built and their accuracy was verified by experimental results. Parameter studies were carried out to investigate the effect of geometric and loading parameters on the bending properties of the Diamond LCSs by the finite element model. The results show that Diamond LCS has a stable “V” deformation pattern under a three-point bending load. In the range of relative density (*RD*) = 15–30%, the higher the *RD*, the better the lateral bending performance of the Diamond LCS structure. The larger the variation radial coefficient, the higher the lateral load-carrying capacity of the structure. The smaller the loading angle of the punch, the better the lateral bending performance of the Diamond LCS structure. However, if the loading angle is too small, the structure is prone to large torsional deformation, and the deformation tends to destabilize. The increase in punch diameter effectively improves the deformation pattern and bending energy absorption characteristics of the structure. The smaller the span of the cylindrical support, the better the bending energy absorption characteristics of the structure.

## 1. Introduction

Cylindrical shell structures are widely used in the fields of ships, transportation, and aerospace due to their advantages of lightweight, high specific strength, high specific stiffness, and high energy absorption [1,2,3,4]. The studies show that the cylindrical shell structure designed with lattice structure exhibits more excellent mechanical properties compared with the traditional cylindrical shell structure in relevant studies [5,6]. Therefore, the use of lattice structures for the protection design of cylindrical shell structures has important scientific research significance and engineering application value.

To improve the mechanical properties of lattice cylindrical shell structures (LCS), researchers have carried out a lot of research work. For example, Zhang et al. [7] studied the deformation failure mechanism of lattice cylindrical shell structure with non-sib form under compressive load by theoretical analysis and numerical simulation. The finite element results show that the mechanical properties of Kagome and triangular lattice cylindrical shells are better than those of hexagonal lattice cylindrical shells. Chen et al. [8] systematically studied the mechanical properties of triangular and hexagonal lattice cylindrical shells under constant velocity impact, established the theoretical solution model of dynamic platform stress of lattice cylindrical shells, and summarized the influence of relative density gradient on energy absorption of lattice cylindrical shells and lattice sandwich cylindrical shells. Belardi et al. [9] proposed an anisogrid composite lattice shell structure with stiffness and studied their buckling failure load and mode using a discrete approach. Then an optimization procedure was proposed based on the NSGA-II and the optimal results fulfilling all the constraints were obtained. Wang et al. [10] conducted the axial compression tests on cylindrical shells containing cracks and built the finite element (FE) models to examine their elastic and elastic-plastic buckling load and modes. They found that the buckling load increases with the increase in crack inclination. An et al. [11] proposed a novel lattice cylindrical shell structure, and its vibration transmission and load-bearing properties were examined by simulations and tests. The results indicate that the proposed structures exhibit excellent vibration isolation performances. Zhou et al. [12] studied the collapse mechanism of the LCSs using a 3D FE model. It is found that the LCSs exhibit a three-stage collapse mechanism “double-diagonal -members-failure-belt” under severe earthquakes.

Recently, the triply periodic minimal surfaces inspired by natural materials have drawn more attention due to their excellent mechanical and energy absorption properties [13,14,15]. For example, Zhang et al. [16] studied the mechanical properties and energy absorption properties of three TPMS structures (Primitive, Diamond, and Gyroid) and Body-Centered Cubic (BCC) lattice structures under compressive load through experiments. Experimental results show that the compression performance of the TPMS structure is better than that of the BCC lattice structure. Sokollu [17] studied the mechanical properties of three planar TPMS structures (Primitive, Diamond, and Gyroid) and two truss lattice structures (BCC and face-centered cube) through tensile and compressive experiments. The experimental results show that the strength of the TPMS structure is twice that of the truss lattice structure under tensile load, and the TPMS structure shows better energy absorption performance than the truss lattice structure.

TPMS lattice is introduced into the design of lattice cylindrical shells to further improve the mechanical properties and energy absorption characteristics. For instance, Wang et al. [18] proposed a new type of Gyroid lattice cylindrical shell structure. The Gyroid-TPMS lattice structure is transformed into a lattice cylindrical shell structure by the mapping method. The crushing performance of the Gyroid lattice cylindrical shell is verified by axial compression test and finite element analysis. The simulation results show that the energy absorption performance of the Gyroid lattice cylindrical shell is better than that of triangular and hexagonal lattice cylindrical shells. Cao et al. [19,20] investigated the influence of printed defects on the mechanical properties of the TPMS LCS using experimental tests and numerical simulations. It is found that the geometric defects have significant effects on the mechanical properties of the TPMS LCS. 20. Szatkiewicz et al. [21] investigated the mechanical and energy absorption abilities of the mapped TPMS LCSs which were fabricated by additive manufacturing with 316L stainless steel. Zhu et al. [22] proposed a mapping method to construct the D-LCS. The energy absorption properties were studied using experimental and numerical methods. The results show that the proposed D-LCS exhibits excellent energy absorption performance. Then, a TPMS-filled cylinder shell structure was proposed and its energy absorption properties were investigated by LS-DYNA [23]. The optimal D-TPMS-CS structure was derived using the multi-objective particle swarm optimization algorithm and surrogate models.

Most of the above-mentioned works are concerned with the mechanical and energy absorption properties of the TPMS LCS under axial compression loading. To the author’s best knowledge, the bending performance of the TPMS LCS has not been reported to date.

Therefore, the finite element model of TPMS lattice cylindrical shell structure under three-point bending is established and the accuracy of the model is verified using experimental results. The influence of structural parameters on the bending performance of TPMS lattice cylindrical shell structure was studied using the finite element model.

## 2. Materials and Methods

### 2.1. Structural Design of TPMS Lattice Cylindrical Shell

The Diamond TPMS surface can be defined by the level set equations [16,17,22],

(1) $$\begin{array}{c} \varphi \left(x,y,z\right)\equiv \sin\left(\omega x\right)\sin\left(\omega y\right)\sin\left(\omega z\right)+\cos\left(\omega x\right)\cos\left(\omega y\right)\cos\left(\omega z\right)+\\ \sin\left(\omega x\right)\cos\left(\omega y\right)\cos\left(\omega z\right)+\cos\left(\omega x\right)\cos\left(\omega y\right)\sin\left(\omega z\right)=c \end{array}$$

where φ(x,y,z) is the function to define the Diamond TPMS surface, x, y, and z are spatial coordinates, ω=2π/L, and L is the unit cell length, c is a constant that controls the spatial position of the Diamond surfaces. The sheet-diamond lattice is built by filling the volume enclosed by two Diamond surfaces (φ=±c). The relative density (RD) of the sheet-diamond lattice is defined by $RD=V_{Diamond}/V_{cube}$, where $V_{Diamond}$ is the volume of the sheet-diamond lattice, and $V_{cube}$ is the volume of the cube with the length of L.

**Figure 1:**
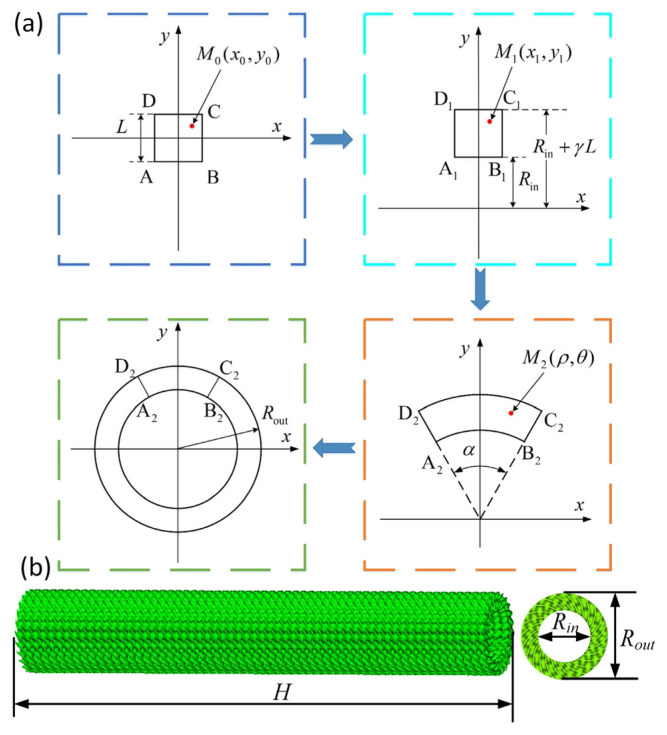
(**a**) The construction process of the LCS structure using TPMS surfaces. (**b**) The proposed Diamond LCS structure.

The geometric model of the Diamond LCS structure is obtained by the method proposed by Zhu in [22], as shown in Figure 1a. Two coefficients, i.e., radial variation coefficient (γ), and mapping angle (α) are introduced to control the structural shape of the Diamond LCS. γ is defined as $\gamma =(R_{\mathrm{out}}-R_{\mathrm{in}})/L$, $R_{out}$ is the outer diameter of the Diamond LCS, and $R_{in}$ is the inner diameter of the Diamond LCS. α is defined as α=2π/n, n=1, 2, 3,⋯. The construction of the Diamond LCS structure is described as follows: (1) The unit cell of the Diamond is placed on the XOY plane; (2) The unit cell of the Diamond is transformed using $x_{1}=x_{0}$, $y_{1}=y_{0}+R_{\mathrm{in}}+\gamma \cdot L/2$; (3) The unit cell of the Diamond is transformed to the polar coordinate system using $\rho =y_{1}$, $\alpha =x_{1}\cdot \alpha /L$; (4) The complete Diamond LCS can be obtained by circumferentially arraying the sector-shaped cylindrical region.

An in-house program was used to obtain the stl file for the Diamond LCS structure using Matlab software (version R2022b), Figure 1b gives the Diamond LCS structure built by the above method, where H is the height of the Diamond LCS.

### 2.2. Finite Element Model

**Figure 2:**
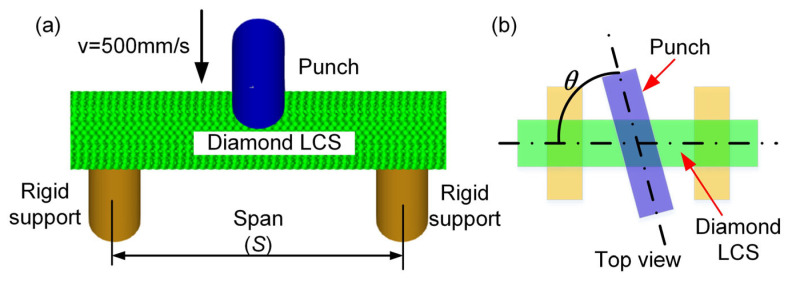
(**a**) FE model of Diamond LCS under three-point bending; (**b**) illustration of the loading angle.

The numerical model of the three-point bending of the Diamond LCS structure is established using explicit nonlinear dynamics software LS-DYNA (version 971 R4.2), as shown in Figure 2a. The Diamond LCS is symmetrically arranged on two cylindrical supports, and a punch is located at the center of the test structure with the loading angle (θ), which is defined as the angle between the punch and the Diamond LCS, as shown in Figure 2b. The Diamond LCS structures are modeled using Belytschko–Tsay shell elements with reduced integration. The punch and the supports are modeled by the shell elements with rigid material. The mesh size is adapted to 0.4 mm using the convergence tests. The diameter of the two supports is 20 mm, the diameter of the punch is D, and the span (S) of the two supports is set to 110 mm. The punch compresses the lattice cylindrical shell structure downward at a speed of 500 mm/s, and the termination displacement is set to 50 mm.

**Figure 3:**
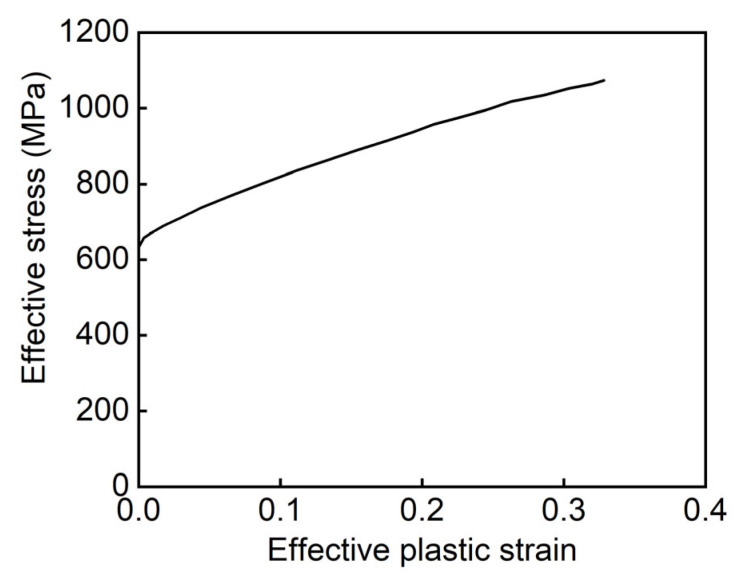
Effective stress-plastic strain curves of the 316L stainless steel [22].

The constitutive behaviors of the base material were simulated using the piecewise linear plasticity material MAT24. Diamond LCS is made of 316L stainless steel, and its mechanical properties are listed in Table 1 and Figure 3 [22]. The contact between Diamond LCS, punch, and support is set as an automatic surface-to-surface contact algorithm, and the self-contact of Diamond LCS is simulated by an automatic single surface contact algorithm, which avoids the penetration of cells in the bending process of the structure. The static and dynamic friction coefficients are set to 0.3 for all contact settings [22,23].

### 2.3. Crashworthiness Evaluation Index

To evaluate the crashworthiness properties of the Diamond LCS, energy absorption (EA), specific energy absorption (SEA), peak breaking force (PCF), and impact force efficiency (CFE) are used in this study [24,25,26,27].

**Figure 4:**
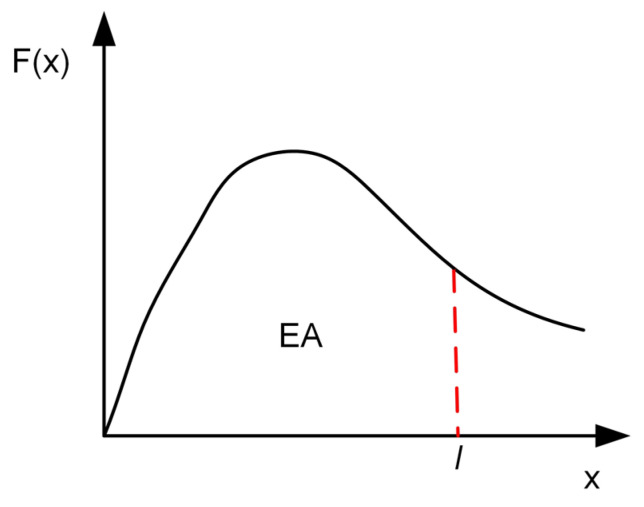
Illustration of the energy absorption properties of the energy absorber.

As shown in Figure 4, Energy Absorption (EA) is used to measure the energy absorption capacity of the whole energy-absorbing structure, and its mathematical expression is

(2) $$EA=\int_{0}^{l}F(x)dx,$$

where, F(x) is the instantaneous collision force at a given crushing distance l.

Specific Energy Absorption (SEA) can be used to compare the energy absorption capacity per unit mass of different structures, and the mathematical expression of SEA is

(3) $$\mathrm{SEA}=\frac{\mathrm{EA}}{m},$$

where, m is the mass of the energy-absorbing structure. The higher the SEA, the better the energy absorption capacity of the energy-absorbing structure.

Peak Crushing Force (PCF) is the maximum value of the collision force in the whole collision process.

Crushing Force Efficiency (CFE) represents the smoothness of the collision force-displacement curve. CFE is defined as the ratio of Mean Crushing Force (MCF) to PCF

(4) $$\mathrm{CFE}=\frac{\mathrm{MCF}}{\mathrm{PCF}},$$

where MCF is the average value of collision force at a given collision distance, and the calculation formula is

(5) $$\mathrm{MCF}=\frac{\mathrm{EA}}{l},$$

Generally, the larger the CFE value, the better the energy absorption efficiency of the energy absorption structure.

## 3. Results and Discussions

### 3.1. Verification of Finite Element Model

**Figure 5:**
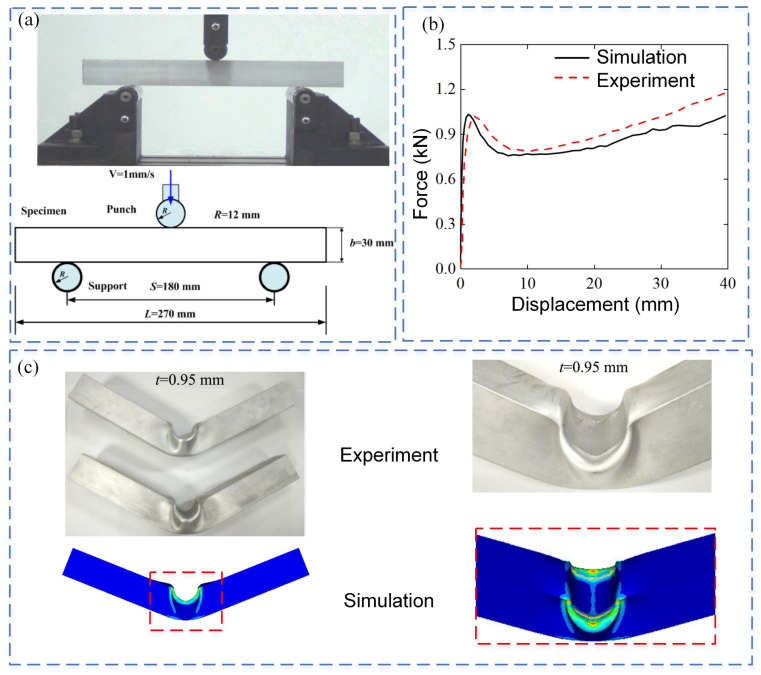
Verification of the numerical model: (**a**) Geometric details of three-point bending of thin-walled square tube in reference [28]; (**b**) Experimental and simulation force-displacement curves of square tube bending; and (**c**) Comparison of the experimental and simulation flexural deformation modes of thin-walled square tubes with wall thickness t = 0. 95 mm.

To verify the reliability of the proposed finite element modeling method, the experimental results of thin-walled square tubes under three-point bending are selected from reference [28]. The geometrical dimensions of the thin-walled square tube are shown in Figure 5a, and the square tube specimen is manufactured with aluminum alloy AA6063-O. The deformation process and force-displacement curves of thin-walled square tubes under three-point bending are simulated using the FE modeling method by LS-DYNA. The square tubes, punch, and supports are meshed by Belytschko-Tsay 4-node shell elements with five integration points, mesh size is used as 1.0 mm. Figure 5b and Figure 5c compare the deformation patterns and mechanical responses between the experimental and simulation results. It can be seen that the experimental results in the literature are quite consistent with the numerical results obtained by simulation.

**Figure 6:**
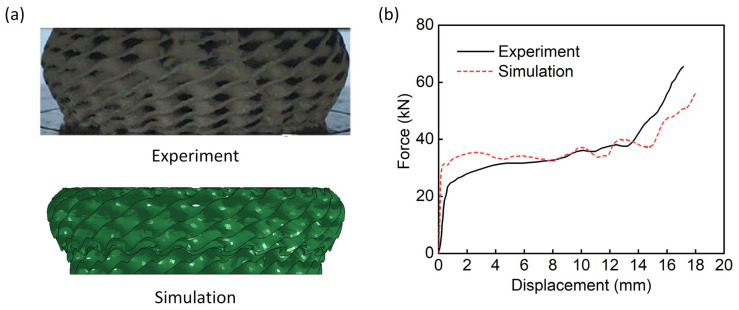
Verification of the numerical model [22]. Comparison of (**a**) the experimental and simulation deformation modes and (**b**) Experimental and simulation force-displacement curves.

To further verify the material model for the proposed finite element modeling method, the reference [22] is selected to keep the base material used in this study is same as the selected reference. The Diamond LCS has a height of 24 mm, an inner radius of 9 mm, and an outer radius of 15 mm. The deformation process and force-displacement curves of the Diamond LCS are simulated using the FE modeling method. Figure 6a,b compare the deformation patterns and mechanical responses between the experimental and simulation results. It can be seen that the experimental results in the literature are quite consistent with the numerical results obtained by simulation. These results indicate that the finite element modeling method proposed in this paper can be used in the following study of Diamond LCS bending performance.

### 3.2. Parameter Study

In this section, the energy absorption characteristics of Diamond LCS structures with different structural parameters (relative density, radial variation coefficient, and mapping angle) are analyzed by the finite element model.

#### 3.2.1. Effect of Relative Density

To investigate the influence of RD on the bending properties of Dimond LCS, four Diamond LCS with different RD (0.15, 0.20, 0.25, 0.30) were studied by finite element analysis. All the Diamond LCSs used the same geometric dimensions and loading conditions: γ = 1, α=30°, θ=90°, D=20 mm, and S=110 mm.

**Figure 7:**
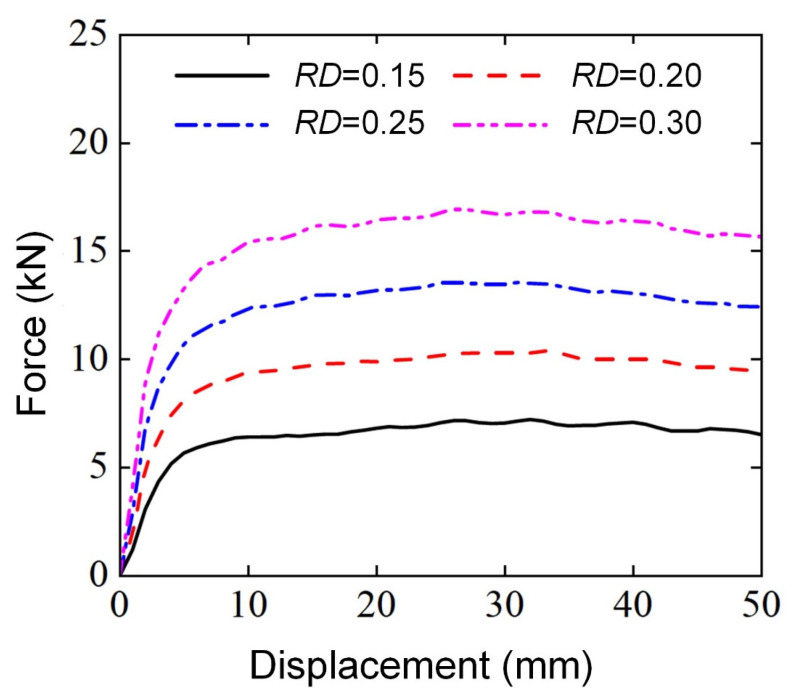
Force-displacement curves of Diamond LCS with different RD.

Figure 7 presents the load force-displacement curves of Diamond LCS structures with different relative density RD (0.15, 0.20, 0.25, 0.30) under a three-point bending load. Firstly, it can be found from Figure 7 that Diamond LCS structures with different RD have similar evolution trends under a three-point bending load, that is, with the compression of punch, the load force of Diamond LCS structure first increases sharply, and then the load force presents a high and long platform stage until the deformation ends. The PCFs and platform force of the Diamond LCS structure increase rapidly with the increase in RD, which not only means that the stiffness and strength of the structure under bending load are greatly improved but also the Diamond LCS structure with larger RD has better bending energy absorption potential. Secondly, the Diamond LCS structure can smoothly transition to the platform stage after the end of the elastic deformation stage, instead of going through a hump-like change process of rising-falling like common thin-walled tubes in reference [29]. The reason may be that the TPMS structure has the characteristics of uniform stress and is beneficial for reducing stress concentration. This also confirms the advantages of TPMS structure in bending and energy absorption.

**Figure 8:**
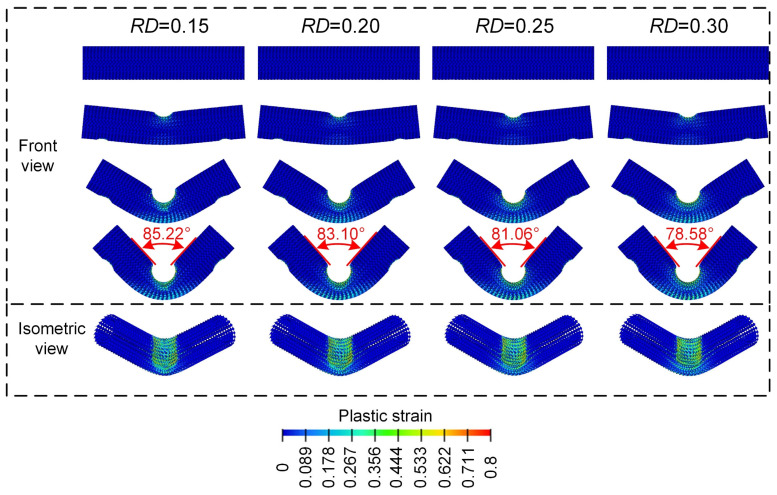
Bending deformation modes of Diamond LCS with different RD.

From Figure 8, the final deformation modes of the Diamond lattice cylindrical shell structure under a three-point bending load all exhibit a similar “V” shape. With the increase in the contact surface between the punch and the Diamond LCS structure, the concave deformation zone near the cylindrical support is fixed and formed. Then the plastic strain on the inner side of the Diamond LCS structure and the plastic deformation on the outer side is more obvious, which indicates that more materials participate in the energy absorption process. This phenomenon makes the Diamond LCS structure have higher energy absorption efficiency. With the increase in RD, the “V” angle of the structure decreases gradually. Nevertheless, the deformation of Diamond LCS with different RD is almost unchanged.

**Figure 9:**
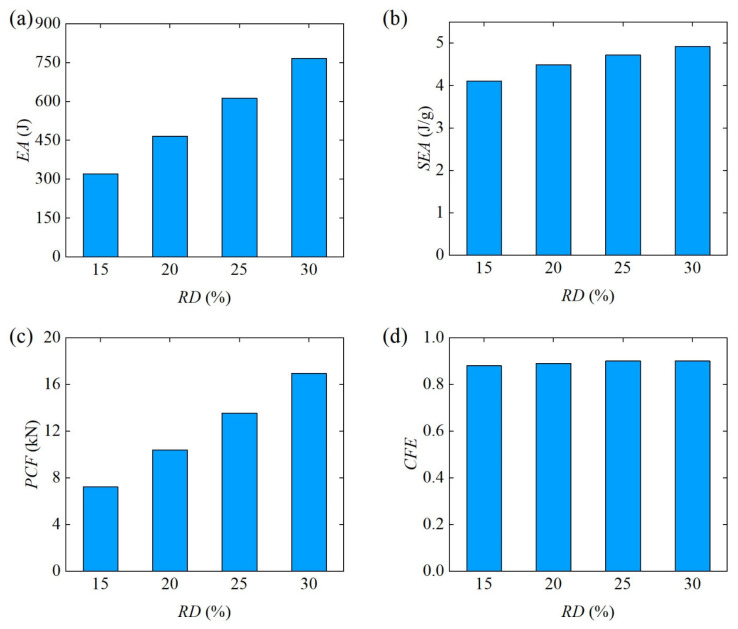
Crashworthiness of the Diamond LCS with different RD: (**a**) EA, (**b**) SEA, (**c**) PCF, and (**d**) CFE.

Finally, the bending energy absorption characteristics of Diamond LCS structures with different RD are further compared in Figure 9. The EAs and SEAs of Diamond LCS increased linearly with the increase in RD. That is, the larger the RD, the better the energy absorption of Diamond LCS. As shown in Figure 9c,d, PCF of the Diamond with different RD has a similar change rule to EA, and it also increases monotonically with the increase in the RD. Although the larger the RD, the larger the PCF. The CFE of Diamond LCS was not sensitive to RD, and the CFE of all configurations remained at a relatively stable level (about 0.89). In short, the larger the RD, the better the lateral bending performance of Diamond LCS.

#### 3.2.2. Effect of Radial Variation Coefficient (γ)

In order to investigate the influence of the radial variation coefficient (γ), the deformation modes and lateral flexural properties of four different Diamond LCS configurations (γ=0.5, 1.0, 1.5, 2.0) were studied by finite element analysis. All the Diamond LCSs used the same geometric dimensions and loading conditions: RD = 0.2, α=30°, θ=90°, D=20 mm, and S=110 mm.

**Figure 10:**
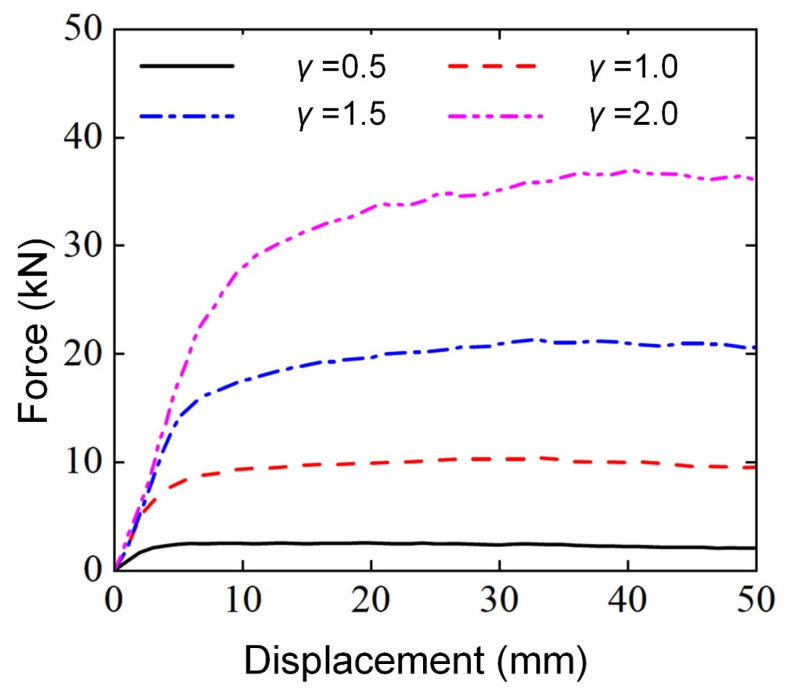
Force-displacement curves of Diamond LCS with different γ.

Figure 10 shows the force-displacement curves and deformation modes of Diamond LCS with different radial variation coefficients under three-point bending loads. It can be seen from Figure 10 that the bending load curves with different radial variation coefficients have similar trends, and the elastic peak value and platform force of the structure increase rapidly with the increase of γ. The greater the γ, the more gentle the transition from the elastic deformation stage to the platform stage for the Diamond LCS.

**Figure 11:**
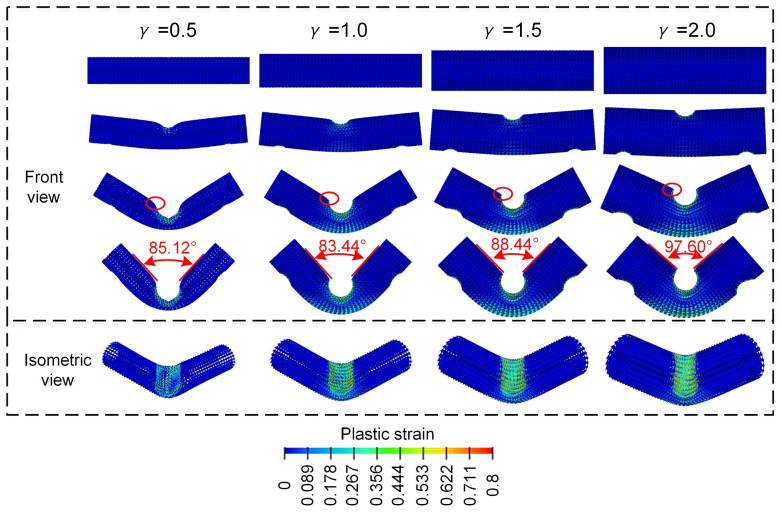
Bending deformation modes of Diamond LCS with different γ.

As shown in Figure 11, all Diamond LCSs have a similar “V” deformation pattern, i.e., plastic deformation and strain are symmetrically distributed along the punch intrusion centerline. Compared with other configurations, the Diamond LCS with γ=0.5 configuration has larger cross-section deformation and the grooves formed by punch invasion even tend to spread towards both sides of the structure, which greatly increases the risk of structural failure. On the contrary, with the increase of γ, more material on the side of the punch participates in energy absorption, and there is an obvious plastic deformation area on the outside. These results show that the larger the γ, the more lateral bearing capacity of the Diamond LCS.

**Figure 12:**
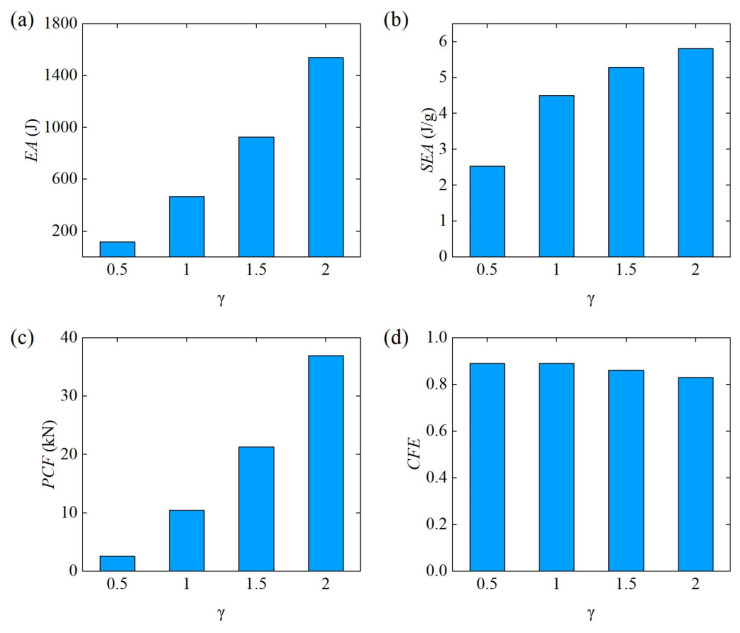
Crashworthiness of the Diamond LCS with different γ: (**a**) EA, (**b**) SEA, (**c**) PCF, and (**d**) CFE.

The bending energy absorption characteristics of different Diamond LCS configurations can be compared more intuitively through Figure 12. From Figure 12a,b, it can be found that the energy absorption of the Diamond LCS structure increases rapidly with the increase of γ. Among them, EA and SEA show a trend of almost exponential growth. Similar to EA, the PCF of Diamond LCS also increased monotonously with the increase of γ. The CFE of different Diamond LCS structures remained at a high level (all above 0.8), as shown in Figure 12d. This shows that the larger γ, the better the lateral flexural performance of the Diamond LCS structure.

#### 3.2.3. Effect of Mapping Angle (α)

In order to investigate the effect of mapping angle (α) on the deformation modes and lateral flexural properties of Diamond LCS, four Diamond LCS with different mapping angles (α=15°, α=30°, α=45°, α=60°) were studied by finite element analysis. All the Diamond LCSs used the same geometric dimensions and loading conditions: RD = 0.2, γ=1.0, θ=90°, D=20 mm, and S=110 mm.

**Figure 13:**
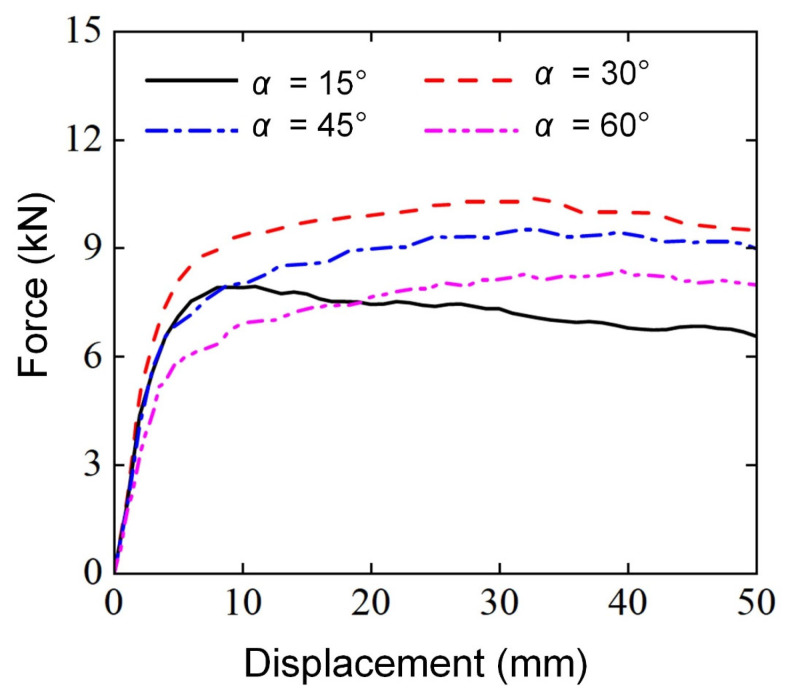
Force-displacement curves of Diamond LCS with different α.

Figure 13 shows the force-displacement curves and deformation modes of Diamond LCS with different mapping angles (α=15°, α=30°, α=45°, α=60°) under three-point bending loads. It can be found that the load-bearing curves of all Diamond LCS structures show a two-stage trend of elastic deformation and platform plastic force. In the range of α = 30–60°, the elastic peak value and platform force decrease with the increase of α. However, the bending force for the Diamond LCS with α=15° slowly decreases in the plastic stage. Diamond LCS structure with α = 30 has the best lateral bearing characteristics.

**Figure 14:**
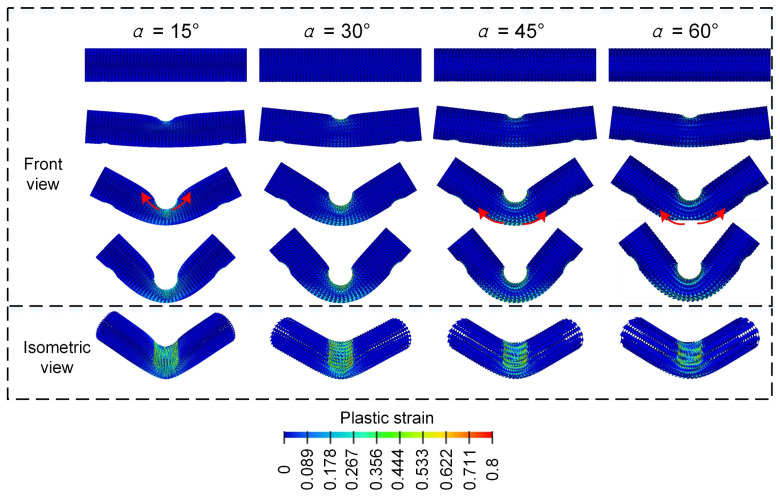
Bending deformation modes of Diamond LCS with different α.

Figure 14 compares the deformation processes and bending deformation modes of the Diamond LCS structures with different α. For Diamond LCS structure with α=30°, α=45°, α=60°, the outer cells of the configuration participate in more plastic deformation and spread the load toward both sides of the centerline as the punch goes deeper, which can avoid the stress being concentrated at the lowest point (front view). On the contrary, because of the thin wall thickness of the Diamond LCS structures with α=15°, the inner cells first undergo plastic deformation and spread along the two ends of the structure.

**Figure 15:**
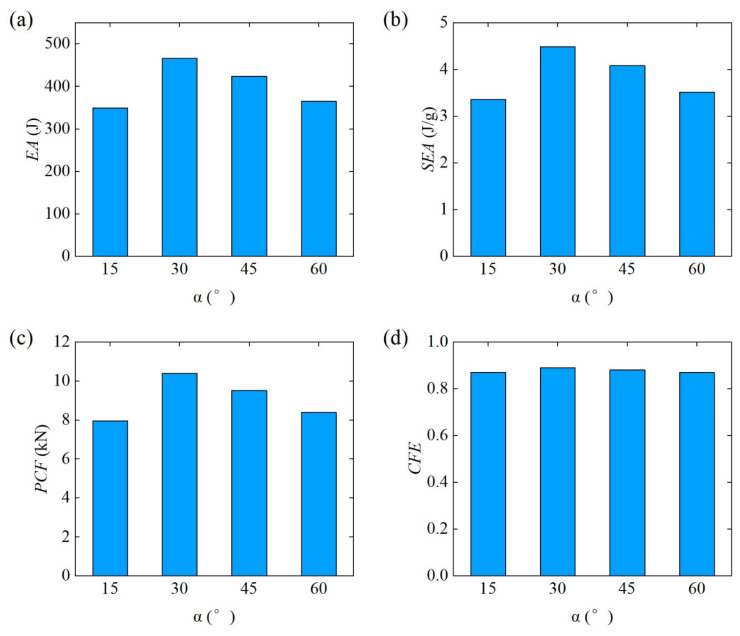
Crashworthiness of the Diamond LCS with different α: (**a**) EA, (**b**) SEA, (**c**) PCF, and (**d**) CFE.

Figure 15 further compares the bending energy absorption characteristics of Diamond LCS structures with different α. In conjunction with Figure 14, the Diamond LCS structure with α=15° has the worst energy absorption performance, and the Diamond LCS structure with α=15° has the best lateral bending performance. For energy absorption, EA and SEA of the Diamond LCS structure with α=30° are 33.38%, 10.06%, and 27.77% higher than those of other configurations, respectively. At the same time, the configuration has the highest PCF (10.4 kN) and the highest impact force efficiency (CFE = 0.89).

#### 3.2.4. Effect of Loading Angle (θ)

Under the actual lateral impact condition, the energy absorption protection structure may encounter impact in any direction. Therefore, the influence of different loading angles (θ = 90°, 75°, 60°, 45°) on the lateral flexural properties of Diamond LCSs was studied through finite element analysis. All the Diamond LCSs used the same geometric dimensions and loading conditions: RD = 0.2, γ=1.0, α=30°, D=20 mm, and S=110 mm.

**Figure 16:**
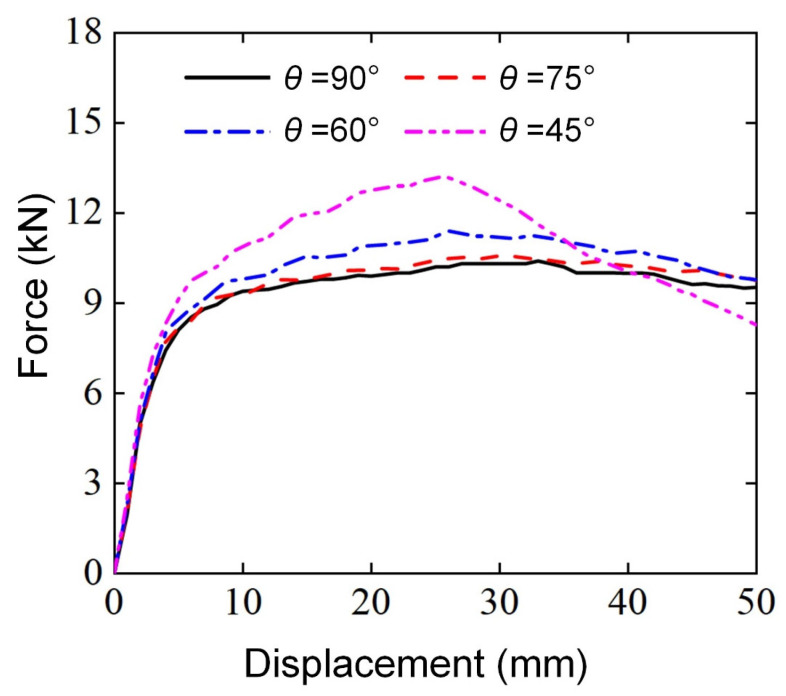
Force-displacement curves of Diamond LCS with different θ.

Figure 16 shows the bending load force-displacement curve of Diamond LCSs at different loading angles. All the Diamond LCSs have similar force-displacement curve deformation trends and the elastic peak value and platform force increase with the decrease in loading angle θ. Combined with the final deformation mode in Figure 14, it can be found that with the decrease in loading angle, the effective plastic deformation area between the punch and Diamond LCS increases, and the bending force of Diamond LCS naturally increases. At the same time, the decrease in loading angle aggravates the relative sliding between the punch and Diamond LCS, and even obvious torsional deformation appears in the configuration.

**Figure 17:**
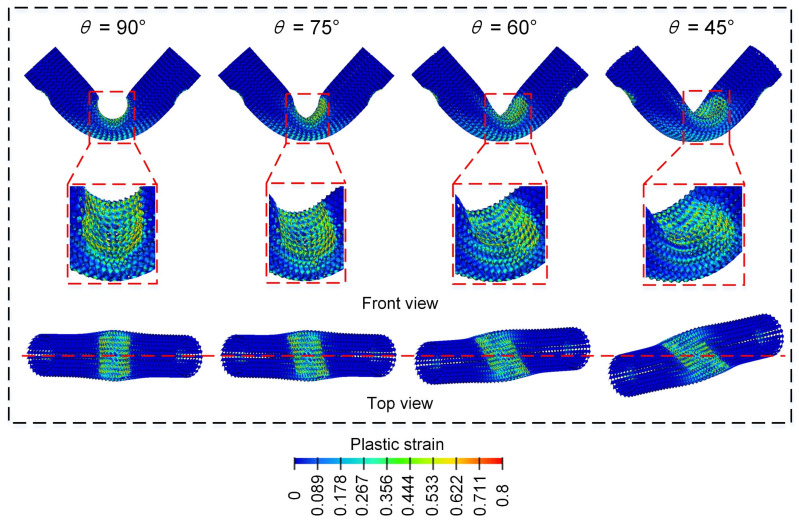
Bending deformation modes of Diamond LCS with different θ.

From Figure 17, we can find that more materials are involved in energy absorption with the decrease of θ. The Diamond LCS with θ = 45° has the highest bending force in the early stage, while the bending force decreases in the later stage due to most of the Diamond LCS structures being yielded.

**Figure 18:**
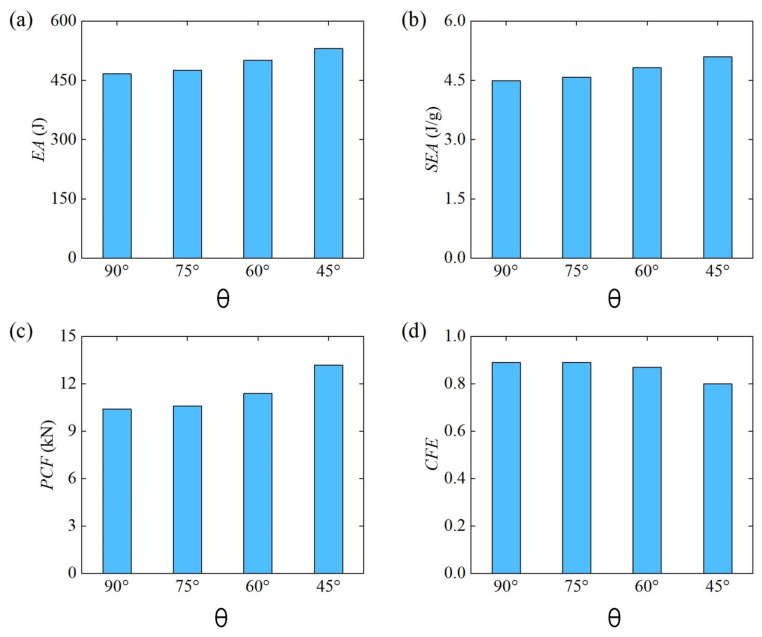
Crashworthiness of the Diamond LCS with different θ: (**a**) EA, (**b**) SEA, (**c**) PCF, and (**d**) CFE.

Figure 18 further shows the comparison of energy absorption characteristics of Diamond LCS under different loading angles. The results show that the energy absorption capacity and efficiency of Diamond LCS increase with the decrease in loading angle. When the punch is loaded downward perpendicular to the structural axis, the energy absorption effect of this configuration is the worst. As shown in Figure 18c, the PCF of the Diamond LCS also increases with the decrease in the loading angle. At the same time, the impact force efficiency decreases monotonously with an increase in the loading angle, while the CFE is maintained at a relatively high level.

#### 3.2.5. Effect of Punch Diameter (D)

In previous studies on the three-point bending performance of thin-walled structures, it was found that the diameter of the punch also has an impact on the deformation mode of structures [28,29]. Therefore, four numerical models with different punch diameters (D=5, 10, 20, 30 mm) were established using the finite element method to study the influence of punch diameters on the lateral flexural properties of Diamond LCSs. All the Diamond LCSs used the same geometric dimensions and loading conditions: RD = 0.2, γ=1.0, α=30°, θ=90°, and S=110 mm.

**Figure 19:**
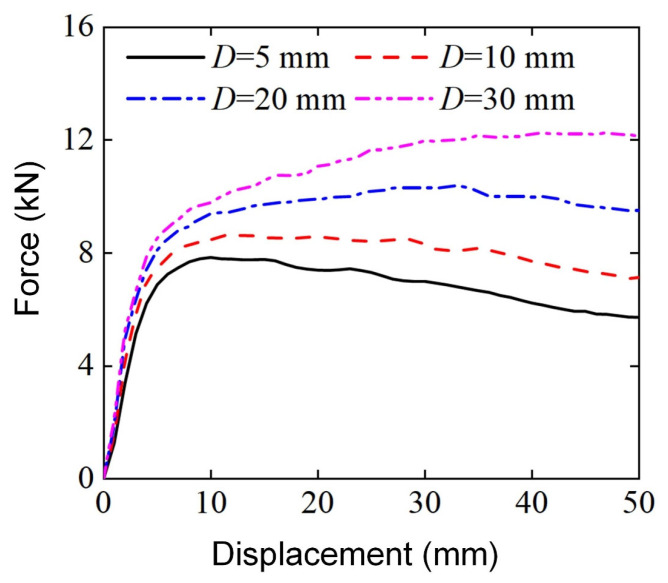
Force-displacement curves of Diamond LCS with different D.

**Figure 20:**
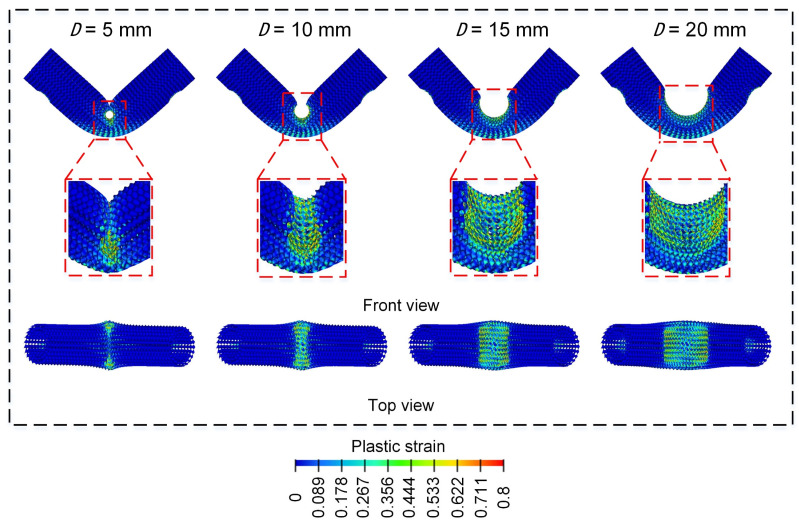
Bending deformation modes of Diamond LCS with different D.

Figure 19 shows the bending force-displacement curve of Diamond LCS for four different punch diameters. All curves show a two-stage change trend, and the elastic peak value increases with the increase in punch diameter. The difference is that the bending force of D=5, 10 mm continues to decrease after the elastic peak value, while the load force of D=20 mm shows a platform stage after the elastic peak value. The bending force of D=30 mm continues to increase after the elastic peak value. Combined with the final deformation mode in Figure 20, it can be found that the punch diameter has a significant influence on the deformation mode. With the increase in punch diameter, the effective deformation area inside Diamond LCS is enlarged and the deformation is more serious. When D≤20 mm, the entire punch is almost embedded in the Diamond LCS structure.

**Figure 21:**
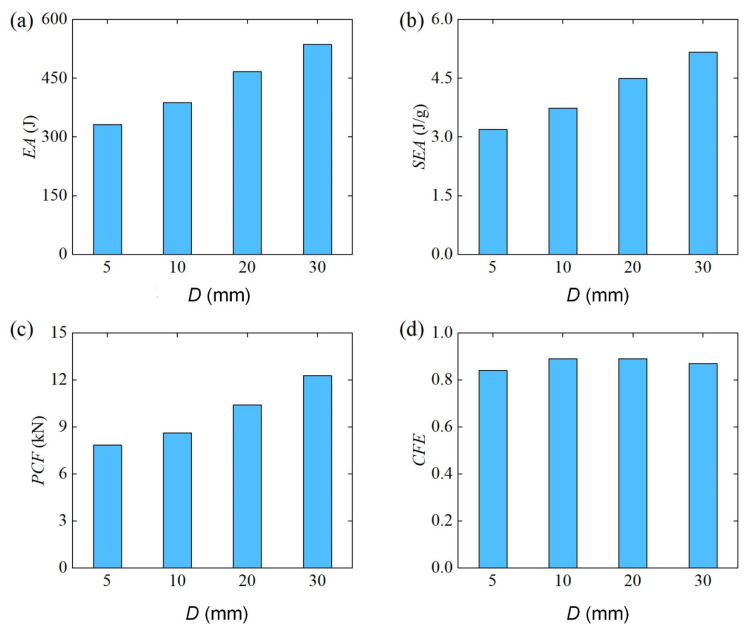
Crashworthiness of the Diamond LCS with different D: (**a**) EA, (**b**) SEA, (**c**) PCF, and (**d**) CFE.

Figure 21 further shows the comparison results of energy absorption characteristics of Diamond LCS under different punch diameters. The results show that EA, SEA, and PCF of Diamond LCS increase monotonously with the increase in punch diameter. At the same time, its impact force efficiency has no obvious change after the punch diameter and generally maintains a high level.

#### 3.2.6. Effect of Span (S)

To investigate the influence of bending span (S), the deformation mode and lateral bending behavior of Diamond lattice cylindrical shells with different spans S (130, 110, 90, 70 mm) were analyzed by finite element method. All the Diamond LCSs used the same geometric dimensions and loading conditions: RD = 0.2, γ=1.0, α=30°, θ=90°, and S=110 mm.

**Figure 22:**
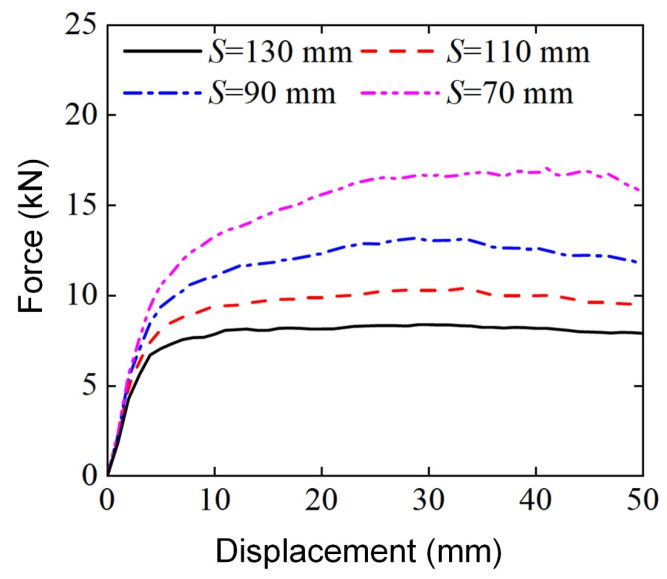
Force-displacement curves of Diamond LCS with different S.

**Figure 23:**
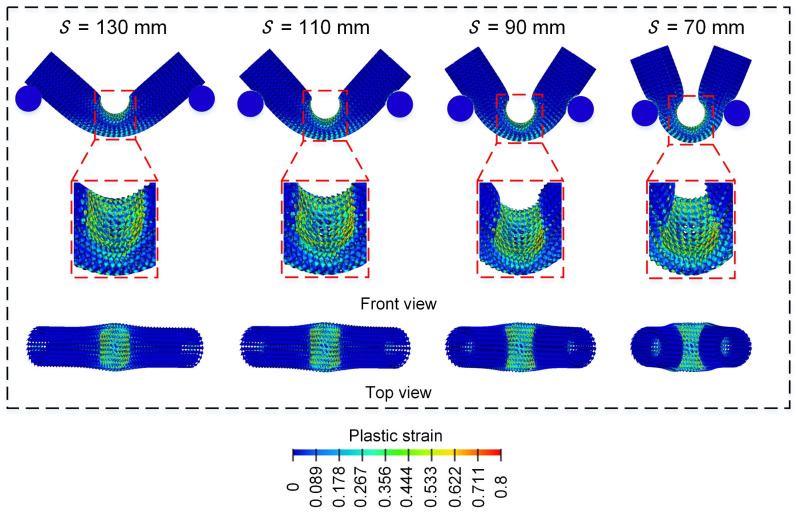
Bending deformation modes of Diamond LCS with different S.

Figure 22 shows the bending load force-displacement curves of Diamond LCSs at different spans. All Diamond LCSs exhibit load characteristics with similar evolution trends, that is, the force goes through a platform stage with a long displacement after the elastic deformation stage until the end of deformation. The elastic peak value and platform force of Diamond LCS increase with the decrease in span. Combined with the final deformation pattern diagram in Figure 23, it can be found that with the decrease in span, the curvature of bending deformation of the Diamond LCS structure increases. At the same time, the effective plastic deformation area inside and outside the Diamond LCS indentation increases, and more materials are involved in energy absorption.

**Figure 24:**
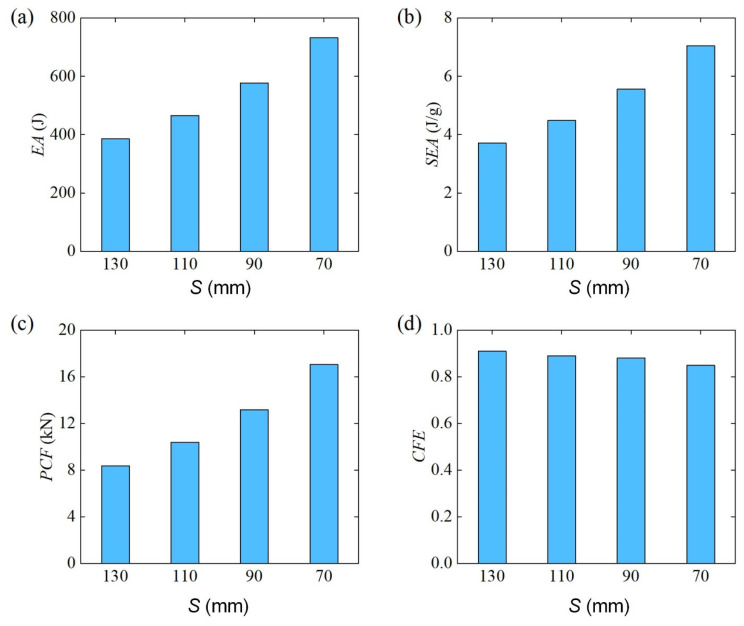
Crashworthiness of the Diamond LCS with different S: (**a**) EA, (**b**) SEA, (**c**) PCF, and (**d**) CFE.

Figure 24 further shows the comparison results of energy absorption characteristics of Diamond LCS at different spans. The results show that the energy absorption and the maximum impact force of the Diamond LCS structure increase with the decrease in the span. At the same time, the CFE of Diamond LCS structures decreases monotonously with the span, while the CFE maintains a high-level CFE≥0.85.

## 4. Conclusions

In this paper, the bending performances of the Diamond LCS structures were investigated through the finite element model, and the influence of different structural parameters on the bending performance of Diamond LCS structures was studied. The conclusions are as follows.

(1) All the Diamond LCS structures exhibit a stable “V” deformation mode under a three-point bending load.
(2) In the range of *RD* = 15–30%, the larger the RD, the better the lateral flexural performance of the Diamond LCS structure. In the range of γ=0.5–2, the larger the radial variation coefficient, the better the lateral bearing capacity of the structure.
(3) The smaller the punch loading angle, the better the lateral bending performance of the Diamond LCS structure. However, if the loading angle is too small, the structure is prone to large torsional deformation, and the deformation tends to be unstable.
(4) The bending energy absorption characteristics of Diamond LCS structures are effectively improved with the increase in punch diameter. The smaller the span, the better the bending energy absorption characteristics of the structure.

## Figures and Tables

**Table 1:** Material properties of 316L stainless steel [22].

Parameter	Value
Density (g/cm^3^)	7.98
Young’s modulus (GPa)	190 ± 3
Yield stress (MPa)	633 ± 9
Ultimate stress (MPa)	1074 ± 38
Poisson’s ratio	0.3

## Data Availability

The original contributions presented in this study are included in the article. Further inquiries can be directed to the corresponding author.
